# Values for preventing influenza-related morbidity and vaccine adverse events in children

**DOI:** 10.1186/1477-7525-3-18

**Published:** 2005-03-21

**Authors:** Lisa A Prosser, Carolyn Buxton Bridges, Timothy M Uyeki, Virginia H Rêgo, G Thomas Ray, Martin I Meltzer, Benjamin Schwartz, William W Thompson, Keiji Fukuda, Tracy A Lieu

**Affiliations:** 1 Department of Ambulatory Care and Prevention, Harvard Medical School and Harvard Pilgrim Health Care, 133 Brookline Ave., 6th floor, Boston, MA, USA; 2 Center for Child Health Care Studies, Harvard Pilgrim Health Care, Boston, MA, USA; 3 National Immunization Program, Centers for Disease Control and Prevention, Atlanta, GA, USA; 4 National Center for Infectious Diseases, Centers for Disease Control and Prevention, Atlanta, GA, USA; 5 Division of Research, Kaiser Permanente, Oakland, CA USA; 6 Division of General Pediatrics, Children's Hospital, Boston, MA, USA

**Keywords:** time-tradeoff, willingness-to-pay, influenza, vaccine, children

## Abstract

**Background:**

Influenza vaccination recently has been recommended for children 6–23 months old, but is not currently recommended for routine use in non-high-risk older children. Information on disease impact, costs, benefits, risks, and community preferences could help guide decisions about which age and risk groups should be vaccinated and strategies for improving coverage. The objective of this study was to measure preferences and willingness-to-pay for changes in health-related quality of life associated with uncomplicated influenza and two rarely-occurring vaccination-related adverse events (anaphylaxis and Guillain-Barré syndrome) in children.

**Methods:**

We conducted telephone interviews with adult members selected at random from a large New England HMO (n = 112). Respondents were given descriptions of four health outcomes: uncomplicated influenza in a hypothetical 1-year-old child of their own, uncomplicated influenza in a hypothetical 14-year-old child of their own, anaphylaxis following vaccination, and Guillain-Barré syndrome. "Uncomplicated influenza" did not require a physician's visit or hospitalization. Preferences (values) for these health outcomes were measured using time-tradeoff and willingness-to-pay questions. Time-tradeoff questions asked the adult to assume they had a child and to consider how much time from the end of their own life they would be willing to surrender to avoid the health outcome in the child.

**Results:**

Respondents said they would give a median of zero days of their lives to prevent an episode of uncomplicated influenza in either their (hypothetical) 1-year-old or 14-year-old, 30 days to prevent an episode of vaccination-related anaphylaxis, and 3 years to prevent a vaccination-related case of Guillain-Barré syndrome. Median willingness-to-pay to prevent uncomplicated influenza in a 1-year-old was $175, uncomplicated influenza in a 14-year-old was $100, anaphylaxis $400, and Guillain-Barré syndrome $4000. The median willingness-to-pay for an influenza vaccination for their children with no risk of anaphylaxis or Guillain-Barré syndrome was $50 and $100, respectively.

**Conclusion:**

Most respondents said they would not be willing to trade any time from their own lives to prevent uncomplicated influenza in a child of their own, and the time traded did not vary by the age of the hypothetical affected child. However, adults did indicate a willingness-to-pay to prevent uncomplicated influenza in children, and that they would give more money to prevent the illness in a 1-year-old than in a 14-year-old. Respondents also indicated a willingness to pay a premium for a vaccine without any risk of severe complications.

## Background

Compared to older children, children under 2 years are at increased risk of influenza-related hospitalization [1-4]. In the United States, it is now recommended that children 6–23 months and household contacts of all children under 2 be vaccinated annually against influenza [5]. Older children without high risk medical conditions are at lower risk of influenza-related complications that may result in hospitalization and are not currently recommended to receive influenza vaccination [1].

Recommendations regarding annual influenza vaccination of children may be aided by considering preference data on the potential costs and benefits of such vaccination. Although some information is available for otitis media and pneumonia (potential influenza illness complications in children), no published studies have quantified preferences, either in terms of quality-adjusted life-years saved or in willingness-to-pay, for preventing influenza and influenza vaccination-related events in children. We conducted a survey to measure the value placed by community members on preventing uncomplicated influenza in children and vaccination-related adverse events including the amount of time required by parents when caring for children with these conditions.

## Methods

### Study sample

Potential respondents were identified via a random sample of all adults enrolled in Harvard Pilgrim Health Care, a large nonprofit health plan in New England, during 2001. Procedures were approved by the Harvard Pilgrim Health Care Human Subjects Committee.

### Survey protocol

Participation in the study consisted of completion of a 30-minute telephone survey. At least one week prior to the telephone interview, potential respondents were mailed materials to refer to during the interview. These included a booklet with written descriptions of uncomplicated influenza and one of two vaccination-related adverse events, either anaphylaxis or Guillain-Barré syndrome (Appendix 1 [see the additional file 1]). The booklet also included instructions for the time-tradeoff and willingness-to-pay questions for the interviewer to review with the respondent during the interview. Respondents were interviewed during April-September, 2002.

The 30-minute closed-ended interview included time-tradeoff questions [6] for uncomplicated influenza and one vaccination-related adverse event. We used the time-tradeoff method to measure preferences because our pilot tests suggested that this method would be more sensitive than other commonly used procedures, such as standard gamble [6]. Time-tradeoff questions asked the respondent to define the amount of time that the respondent would be willing to lose from their own lives so as to prevent uncomplicated influenza in the respondent's child or a hypothetical child (Table 1). The time-tradeoff algorithm used 4 different starting bids followed by three additional bids titrating up. The respondent was then asked the maximum amount of time he or she would be willing to give up within the final interval. Initial bids were 1 week, 2 weeks, 1 month, and 6 months. The time-tradeoff amounts elicited for this study are preference-based measures [7]. Theoretically, the amounts should be lower for less severe health states (respondents willing to give up less time) and higher for more severe health states.

**Table 1:** Sample time-tradeoff and willingness to pay questions

**Introduction:**
When thinking about the following questions:
**Do not include **the costs of medical services or prescription medications. Assume these would already be covered by full insurance even if you don't have this now.
**Do include **the value of preventing the following for you or your child: pain and suffering, inconvenience, and lost time for productive activities (paid work or work in the home) or leisure time.

**Time-tradeoff Question: **Think about what portion of your remaining life, if any, you would be willing to trade off from the end of your life to prevent the flu in your 1-year-old child. You can choose any amount of time in days, weeks, months or years.
Would you be willing to trade off a portion of your remaining life in order to prevent the flu in your 1-year old child? Remember that we are asking you to imagine what portion of YOUR OWN life you would be willing to trade off to prevent influenza in YOUR CHILD.

**Willingness to Pay Question: **Would you be willing to pay some amount of money to prevent an episode of the flu in your one-year-old child?
[If Yes] Would you be willing to pay $200?
[If yes] Would you be willing to pay $400?
[If no] Would you be willing to pay $100?
What is the most you would be willing to pay?

Respondents were also asked two types of willingness-to-pay questions. First, respondents were asked to state the maximum amount of money they would be willing to pay to prevent uncomplicated influenza or one vaccine adverse event (Table 1). Second, they were asked their willingness-to-pay for a specific risk reduction in an influenza vaccination-related adverse event (Table 2). Willingness-to-pay was measured using dichotomous-choice double-bounded questions followed by an open-ended question asking for their maximum willingness-to-pay for both willingness-to-pay questions. Respondents were randomized to four different initial bids to minimize any bias due to the size of the initial bid ("anchoring bias"). For example, initial bids for avoiding uncomplicated influenza in a 1-year-old child were $25, $50, $100, $200. Follow-up bids were double the initial bid if the respondent said yes to the initial bid, and half the initial bid if the respondent said no to the initial bid. See Table 1 for additional details. (For further details on dichotomous-choice questions, see Bateman IJ et al. Economic valuation with stated preference techniques: a manual. Northampton: Edward Elgar Publishing. 2002 or Carson RT. Contingent valuation: A user's guide. *Environ Sci Technol *2000; 34(8):1413–1418].)

**Table 2:** Sample survey question to elicit willingness-to-pay for avoiding a vaccine adverse event

**Vaccine 1**	**Vaccine 2**
• Reduces risk of influenza from 15 in 100 to 3 in 100	• Reduces risk of influenza from 15 in 100 to 3 in 100
• 3 in 10 risk of sore arm after vaccination	• 3 in 10 risk of sore arm after vaccination
• 1 in 100 risk of mild side effects such as fever and muscle aches after vaccination	• 1 in 100 risk of mild side effects such as fever and muscle aches after vaccination
• 1 in 10,000 risk of severe allergic reaction after vaccination	• 1 in 10,000 risk of severe allergic reaction after vaccination
• ***1 in 1 million chance of Guillain-Barré Syndrome after vaccination***	• ***No chance of Guillain-Barré Syndrome after vaccination***
• Costs $10	• Costs $?

Additional questions included how much time had been spent caring for a child with influenza, sociodemographic characteristics, whether the respondent's child had experienced any of the described conditions, and attitude toward childhood vaccination (the respondent was asked how well they agreed with the statement: "the benefits of vaccines are worth the risks" using a 5-item Likert scale from "strongly agree" to "strongly disagree"). To simplify survey design, the order of health state descriptions was not randomized. Respondents were not presented with inconsistencies in their scores, but were asked at the end of the survey if they wished to change any of their answers once the full set of health states had been considered.

### Analysis of survey data

We calculated summary statistics for time-tradeoff and willingness-to-pay amounts, including medians, means, 5th and 95th percentiles, and minimums and maximums. Differences between time-tradeoff and willingness-to-pay responses for avoiding influenza in a 1-year-old as compared with a 14-year-old were evaluated using the sign test for paired observations. Medians were reported along with means because the distributions were skewed toward zero, especially for uncomplicated influenza.

For the time-tradeoff questions, we discounted the time-tradeoff amounts. Respondents were instructed to assume that time would be traded off from the end of their life, therefore we calculated the present value for time-tradeoff amounts using the difference between the participant's age and life expectancy [8] as the timeframe over which to discount, and used a rate of time preference of 3% per year as the discount rate.

The effects of respondent socio-demographic characteristics on time-tradeoff and willingness-to-pay amounts were evaluated using estimated random effects from the generalized linear mixed models (GLMM) version of Poisson regression (SAS v. 8.2, SAS Institute, Cary, NC). This is a two-part model for analyzing data with multiple outcomes from the same respondent. The advantage of using this model for analyzing time-tradeoff and willingness-to-pay data is to account for the correlated responses (in this case the time-tradeoff or willingness-to-pay values) for multiple health states. The underlying assumption being that a respondent's responses across health states will be correlated, i.e., if a respondent has a higher than average time-tradeoff value for one health state they are more likely to have higher than average time-tradeoff values for the other health states. (For additional details, see Burton et al., Tutorial in biostatistics: extending the simple linear regression model to account for correlated responses: an introduction to generalized estimating equations and multi-level mixed modeling. *Statistics in Medicine *1998;17:1261–1291). A Poisson model was selected because time-tradeoff and willingness-to-pay amounts are not distributed normally but are skewed toward zero.

Dependent variables included in the model were age, sex, education (college or more: yes/no), marital status (married: yes/no), income (less than three times poverty level or not), health status (good or better: yes/no), whether the respondent has children under 18 in the household, and whether a child they knew has experienced any of the outcomes described in the survey. We also evaluated the effect of the initial bid by including a dummy variable for each of the four initial bids.

## Results

### Participants

493 letters were mailed to potential respondents to invite them to participate in the survey. Of those invited, 23% could not be contacted to schedule an interview, and 2% were unable to be interviewed in English. Of the 373 remaining potential participants, 32% agreed to participate and completed an interview, 2% scheduled an interview, but refused to participate at the time of the interview, 4% scheduled an interview, but could not be contacted to complete the interview, and 62% refused to participate by either returning an opt-out card or declining (either actively or passively) to schedule an appointment at the time of the follow-up phone call. The overall response rate was 26%. There were more women than men in the sample (Table 3), and respondents were more educated and had higher incomes than the general U.S. population [10,11]. Respondents included 46% who reported having a child who had experienced an episode of influenza, 13% who reported having a child who had experienced a severe allergic reaction, and 1% who reported having a child who had experienced Guillain-Barré Syndrome. This study did not have access to medical records to verify reported events. Seven respondents (6%) who were unable to understand the questions according to interviewer assessment were excluded from further analysis.

**Table 3:** Respondent characteristics (N = 112)

**Characteristics**	**Percent**
Female	58.9%
Mean age, years (SD)	48 (13.8)
Married	66.1%
Have Children Under 18	
Yes	40.2%
No	58.9%
Declined to answer	0.9%
Race	
White	90.2%
Black	6.3%
Asian	3.6%
Educational Attainment	
High school graduate	13.4%
Some college	17.0%
College degree	33.0%
Post graduate training	36.6%
Household income in 2001	
$25,000 or less	5.4%
$25,001–$50,000	17.0%
$50,001–$75,000	25.0%
$75,001–$100,000	13.4%
More than $100,000	26.8%
Declined to answer	11.6%
Did not know	0.9%
Benefits of vaccines are worth the risks	
Strongly agree	44.6%
Agree	39.3%
Neither agree nor disagree	6.3%
Disagree	6.3%
Strongly disagree	0.9%
Did not know	2.7%
Current health (Very good or excellent)	66.9%

### Time-tradeoff and willingness-to-pay

Time-tradeoff amounts increased with the severity of the health state (Table 4). Approximately half of the respondents were not willing to trade off any time from their own life to prevent uncomplicated influenza in a hypothetical child (51% for a 1-year-old child and 60% for a 14-year-old child). The median response for vaccine-induced severe allergic reaction was one month and for Guillain-Barré syndrome 3 years. Discounted mean responses were higher for preventing uncomplicated influenza in a 14-year-old child (41 days) as compared with a 1-year-old (29 days) yet this difference was not significant. An analysis of ranks shows that time-tradeoff responses were consistently higher for uncomplicated influenza in a 1-year-old (despite the higher mean TTO result for uncomplicated influenza in a 14-year-old) (p-value = 0.0596).

**Table 4:** Time-tradeoff amounts for uncomplicated influenza and vaccination-related adverse events

**a. Undiscounted**				
	Median	Mean	5^th ^– 95^th ^percentile	Range (minimum – maximum)
Influenza in 1-year old child*	0	68 days	0 – 1 year	0 – 6 years
Influenza in 14-year old child*	0	86 days	0 – 1 year	0 – 6 years
Severe allergic reaction in 1-year old child	30 days	319 days	0 – 3 years	0 – 20 years
Guillain-Barré Syndrome in 1-year old child	3 years	5 years	0 – 20 years	0 – 25 years
**b. Discounted**				
	Median	Mean	5^th ^– 95^th ^percentile	Range (minimum – maximum)

Influenza in 1-year old child*	0	29 days	0 – 129 days	0 – 2.8 years
Influenza in 14-year old child*	0	41 days	0 – 230 days	0 – 2.8 years
Severe allergic reaction in 1-year old child	11 days	119 days	0 – 1.1 years	0 – 8.3 years
Guillain-Barré Syndrome in 1-year old child	352 days	2.1 years	0 – 7.4 years	0 – 12.1 years

*P-value = 0.0596 using the sign test to assess whether WTP for influenza in a 1-year-old child is greater than WTP for influenza in a 14-year-old.

Median willingness-to-pay amounts ranged from $100 to prevent an episode of influenza in a 14-year-old child to $5,000 to prevent a case of Guillain-Barré syndrome (Table 5). Respondents were also willing to pay a premium of $50 or $100 for hypothetical influenza vaccines that either had no risk of severe allergic reaction or Guillain-Barré syndrome.

**Table 5:** Willingness-to-pay amounts in 2002 dollars for health states

**a. For each health state avoided**				
	Median	Mean	5^th ^– 95^th ^percentile	Range (minimum – maximum)
Influenza in 1-year old child*	175	469	0 – 1500	0 – 10,000
Influenza in 14-year old child*	100	288	0 – 1000	0 – 5,000
Guillain-Barré Syndrome in 1-year old child	5,000	28,579	100 – 100,000	10 – 1,000,000
Severe allergic reaction in 1-year old child	400	4,968	0 – 10,000	0 – 200,000
**b. For risk reductions**				
	Median	Mean	5^th ^– 95^th ^percentile	Range (minimum – maximum)

Influenza vaccine with no risk of Guillain-Barré Syndrome after vaccination	100	341	3 – 2,000	0 – 5,000
Influenza vaccine with no risk of severe allergic reaction after vaccination	50	223	10 – 1,000	0 – 5,000

*P-value < .0001 using the sign test to assess whether WTP for influenza in a 1-year-old child is greater than WTP for influenza in a 14-year-old.

According to the Poisson-based regression analysis, one initial bid (1 week) was associated with higher time-tradeoff responses. None of the other respondent-specific variables (e.g., education, income, health status, or having young children) affected either TTO or WTP results. (Tables 6 and 7)

**Table 6:** Effect of respondent characteristics and initial bids on time-tradeoff amounts

Variable	Type of variable	Impact on TTO	95 % CI	P value
Independent variables:				
TTO amounts^1^				
Dependent variables:				
Baseline^2^	Constant	0.5545	(0.2402, 1.2798)	0.1617
Age^3^				
18–34	Binary	1.0385	(0.4904, 2.1994)	0.9200
35–49	Binary	1.1698	(0.6299, 2.1723)	0.6137
50–64	Binary	1.5448	(0.7921, 3.0129)	0.1959
Female	Binary	0.8629	(0.5926, 1.2565)	0.4342
Education: Some college or less	Binary	1.1579	(0.7537, 1.7789)	0.4966
Not married	Binary	0.9204	(0.5815, 1.4569)	0.7188
Health (Worse than average)	Binary	1.1043	(0.7343, 1.6606)	0.6279
No children under 18 in household	Binary	0.7663	(0.4541, 1.2930)	0.3114
Version (initial bid)^4^				
1 (1 week)	4 binary variables, one for each initial bid	2.8454	(1.7138, 4.7242)	<0.0001
2 (1 month)		1.3331	(0.7943, 2.2374)	0.2694
3 (2 weeks)		1.4705	(0.8741, 2.4739)	0.1415
				
Income (less than 3X poverty level)	Binary	1.0092	(0.5398, 1.8870)	0.9766
Number of disease experienced: None	Binary (0 v. 1 or more)	1.2406	(0.7396, 1.9394)	0.3370

^1 ^Independent variables included for each respondent were uncomplicated influenza in a 1-year-old, uncomplicated influenza in a 14 year-old, and anaphylaxis or Guillain-Barré syndrome. R-squared = 0.2194.

^2 ^Baseline refers to a person over 65 years of age, male, college graduate, married, good health, with income three times or greater than poverty level, children under 18 living in the household, familiar with at least one of the conditions in the survey, and responding to survey version 4 (initial bid of 6 months).

^3 ^Compared to 65 and over.

^4 ^Compared to version 4 (initial bid of 6 months).

**Table 7:** Effect of respondent characteristics and initial bids on willingness-to-pay amounts

Variable	Type of variable	Impact on WTP	95 % CI	P value
Independent variables:				
WTP amounts^1^				
Dependent variables:				
Baseline^2^	Constant	0.9368	(0.3763, 2.3324)	0.8864
Age^3^				
18–34	Binary	1.1458	(0.5055, 2.5974)	0.7401
35–49	Binary	1.6824	(0.8564, 3.3049)	0.1265
50–64	Binary	1.3450	(0.6492, 2.7865)	0.4177
Female	Binary	0.7866	(0.5222, 1.1851)	0.2444
Education: Some college or less	Binary	0.9690	(0.6066, 1.5479)	0.8934
Not married	Binary	0.7606	(0.4610, 1.2551)	0.2773
Health (Worse than average)	Binary	1.1183	(0.7166, 1.7451)	0.6166
No children under 18 in household	Binary	1.1254	(0.6360, 1.9911)	0.6797
Version (initial bid)^4^				
1 ($100)	4 binary variables, one for each initial bid	1.3809	(0.7945, 2.4001)	0.2460
2 ($200)		1.0257	(0.5832, 1.8040)	0.9286
3 ($25)		0.7572	(0.4293, 1.3355)	0.3293
				
Income (less than 3X poverty level)	Binary	0.9811	(0.4957, 1.9416)	0.9554
Number of disease experienced: None	Binary (0 v. 1 or more)	0.6862	(0.4216, 1.1169)	0.1254

^1 ^Independent variables included for each respondent were uncomplicated influenza in a 1-year-old, uncomplicated influenza in a 14 year-old, and anaphylaxis or Guillain-Barré syndrome. R-squared = 0.1725.

^2 ^Baseline refers to a person over 65 years of age, male, college graduate, married, good health, with income three times or greater than poverty level, children under 18 living in the household, familiar with at least one of the conditions in the survey, and responding to survey version 4 (initial bid of $50).

^3 ^Compared to 65 and over.

^4 ^Compared to version 4 (initial bid of $50).

## Discussion

Fewer than half the respondents were willing to trade any time to prevent uncomplicated influenza in a hypothetical child, but many (73%) were willing to give up some time to prevent vaccination-related complications. Most participants indicated a willingness-to-pay to avoid uncomplicated influenza as well as a severe allergic reaction or Guillain-Barré syndrome due to vaccination, but there was substantial variation in the amounts they were willing to pay.

This study did not evaluate willingness-to-pay and time-tradeoff amounts for complications of influenza such as otitis media and hospitalization that should be included in an economic evaluation of influenza vaccine. They were not included because values for these conditions were collected in a previous study conducted on a random sample of adults in the United States. In this study, we found that the median time-tradeoff amount for acute otitis media was 4 days, non-hospitalized pneumonia was 65 days, and hospitalization due to pneumonia was 214 days [12].

For preventing a case of uncomplicated influenza in a 1-year-old and a 14-year-old, 51% and 60% of respondents were not willing to trade any time. For the same health states, far fewer respondents reported zero as their willingness-to-pay to prevent uncomplicated influenza in a 1-year-old (13%) and a 14-year-old (14%). Since the smallest unit respondents could trade was one day in the time-tradeoff questions, respondents that might have been willing to trade a fraction of a day might have responded with zero when the true tradeoff value could have been between 0 and 1 day. Allowing respondents to trade minutes or hours could have resulted in fewer non-zero responses. In this study, respondents were not asked about time periods of less than one day and willingness-to-pay appears to be a more sensitive metric for valuing temporary health states.

The willingness-to-pay results for the safer (hypothetical) vaccines should be interpreted cautiously. There is considerable evidence that people have difficulty valuing small risk reductions and also are willing to pay more in a hypothetical situation [13,14]. In this study, using the responses from the risk reduction questions results in willingness-to-pay estimates orders of magnitude higher than when respondents directly valued the prevention of one case of either event. Differences could be attributable to (1) risk aversion (the second set of values ignores any premium respondents are willing to pay to avoid a risk), (2) overestimation of small probabilities, and/or (3) the voluntary nature of the risk (because parents voluntarily choose to expose their children to vaccines and may feel responsible for bad outcomes associated with them, they may be willing to pay more to reduce that risk than they would in a situation that included a similar risk of experiencing a condition in a way unrelated to any decision or action by the parent). In any case, the values for questions on risk reductions are sufficiently different to cause some concern about the incorporation of probabilities into contingent valuation questions.

There are a number of challenges in measuring the value of health for very young children including the use of parents as proxy respondents, the valuation of temporary health states, and whether or not to include family spillover effects [15-17]. Applying utilities from standardized instruments such as the Health Utilities Index (HUI) or the EQ-5D which were developed to value chronic health states in adults (and children 6 and older in the case of the HUI) are unlikely to be accurate for valuing temporary or transient health states in very young children [15,18]. There is a small but growing body of literature in the area of valuing temporary health states. Alternatives such as the waiting-tradeoff, conjoint analysis, "chained" health states, and other modifications of the time-tradeoff method have been proposed without any clear consensus on a preferred method [19-22]. This study demonstrates the use of a modified time-tradeoff question that differs from that typically used to value chronic health states, in which respondents choose between years of life with and without a stated condition. (For a discussion of the appropriateness of using time-tradeoff questions to elicit utilities for economic evaluations, see Dolan P. Output measures and valuation in health. In: Economic evaluation in health care: Merging theory with practice. Eds: Drummond M, McGuire A. New York: Oxford University Press. 2001.) Approaches similar to the one used in this study have been employed in previous studies [12,23,24], but clearly more research is needed to reach consensus in the field regarding optimal methods for valuing temporary health states in young children.

There has been increasing recognition of family spillover effects (i.e., the effect of one family member's illness on other family members) on health-related quality-of-life. The potential importance of including these effects in economic analyses can be quite significant for illnesses in the very young and the very old [16,17]. Our approach of valuing changes in health-related quality-of-life for both parent and child is consistent with the inclusion of family spillover effects in the economic evaluation. Our study evaluated the tradeoff between life in a parent and a temporary health state in their child. The inclusion of loss of quality-of-life for both parent and child prevents the time-tradeoff amounts from being directly comparable to utility values from generic utility instruments for measuring reductions in quality-of-life for chronic health states, such as the Health Utilities Index [25] or the EQ-5D [26]. Clearly more research will be needed to establish the optimal method for valuing family spillover effects.

The generalizability of these study results are limited by the small sample size and a relatively homogeneous and geographically-limited respondent population whose characteristics differ from those of the general U.S. population. Sampling from the general membership of a large New England HMO resulted in a group of respondents with little variation in income, and that otherwise differed from characteristics of the general U.S. population. For example, more than 75% of respondents had an annual household income greater than $50,000. The response rate was somewhat low, but not atypical for similar telephone surveys. Given these limitations, the results are sufficiently robust to justify a larger study for validation that also included additional uncommon severe outcomes of influenza in children, including encephalopathy and death [27].

The annual probability of a one-year-old experiencing an influenza-related illness is approximately 16% in non-pandemic years [28-37] and varies from 0–23% in a non-pandemic year. The risk of an influenza-related hospitalization is about 3 per thousand for a child at low risk for influenza-related complication [1-3]. In contrast the risk of anaphylaxis from influenza vaccination is estimated at approximately 1 in 4 million [38] based on surveillance from the 1976 swine flu vaccine program. Guillain-Barré syndrome was associated with receipt of swine flu vaccine in 1976 with a risk of 1 per 100,000 persons vaccinated [38], although children were associated with a lower risk of GBS than adults [39], and studies since 1976 have not found a clear association of GBS with influenza vaccination [40,41]. Most studies of an association of Guillain-Barré syndrome with influenza vaccination have been among adults and not children. Economic analyses can provide information useful in comparing the benefits of vaccination with the risks of adverse events.

The variability in preferences and willingness-to-pay observed in this study suggests that different community members may appraise the desirability or cost-effectiveness of influenza vaccination quite differently. The relatively low value many respondents attributed to uncomplicated influenza could provide insight into low coverage rates for influenza vaccination among children. Concern about the safety of vaccination is shown by the premium most respondents were willing to pay for a vaccine with lower risks of adverse events. Information on the costs, benefits, risks, and community preferences can aid policy decisions regarding influenza vaccination.

## Authors' contributions

LAP designed the study, developed the survey, oversaw data collection efforts, directed the analysis, and drafted the manuscript. CBB and TMU participated and contributed data during survey development and critically reviewed the manuscript. VHR supervised data collection and participated in statistical analyses. GTR conducted statistical analyses. MIM participated in survey development and critically reviewed the manuscript. MIM, BS, WWT, and KJ participated in survey development. TAL participated in design and helped draft the manuscript. All authors read and approved the final manuscript.

## Supplementary Material

Additional File 1 Appendix 1. Health State Descriptions for Outcomes Prevented by Influenza Vaccination Click here for file
