# Supplementary material for: Values for preventing influenza-related morbidity and vaccine adverse events in children
Source: Health Qual Life Outcomes. 2005 Mar 21;3:18. doi: 10.1186/1477-7525-3-18 (PMC1083419; doi:10.1186/1477-7525-3-18)
Supplement: Additional File 1 — Appendix 1. Health State Descriptions for Outcomes Prevented by Influenza Vaccination [file 1477-7525-3-18-S1.doc]

**Appendix 1. Health State Descriptions for Outcomes Prevented by Influenza Vaccination[[1]](#footnote-2)**

**1. Influenza in a 1-year-old child**

- Your child is fussy and has a high fever and runny nose for three days, which slowly gets better over the 4th and 5th days.
- Your child is coughing and extremely tired – and does not feel like participating in normal activities like playing.
- After one week, your child completely recovers and has no more problems related to having the flu.

Risk reduction for willingness-to-pay question: To lower the chance of an episode of the flu from 15 in 100 to 3 in 100 during one flu season.

**2. Influenza in a 14-year-old child**

- Your 14-year old teenager is fussy and has a high fever and runny nose for three days, which slowly gets better over the 4th, and 5th days.
- Your child is coughing and extremely tired – and does not feel like participating in normal activities or going to school.
- After one week, your child completely recovers and has no more problems related to having the flu.

Risk reduction for willingness-to-pay question: To lower the chance of an episode of the flu in your teenager from 15 in 100 to 3 in 100 during one flu season.

**3. Severe allergic reaction in a 1-year-old child**

About 5 minutes after receiving a vaccine, your child suddenly

- Develops flushing, sweating, and swelling of the face
- Begins to vomit
- Becomes short of breath and starts to turn blue

The doctor quickly diagnoses this as an allergy to the vaccine and begins medical treatment. This consists of:

- Giving two different shots with medicine to counteract the allergy
- Giving oxygen
- Putting in an intravenous line and giving fluid through the vein

With this treatment, your child quickly recovers and is looking and acting well about 20 minutes after the problem begins. However, because your child has had such a worrisome allergic reaction, he or she has to stay in the hospital overnight for observation. After this, your child is fine and is allowed to go home with no further treatment.

Risk reduction for willingness-to-pay question: Chance of severe allergic reaction after vaccination from 1 in 10,000 to No chance.

## **4. Guillain-Barré Syndrome in a 1-year-old child.**

- Your 1-year old child suddenly experiences severe muscle weakness in his or her legs, then moving up to his or her arms over a period of a few days. Your child also feels cramps, tingling, and numbness in the palms of his or her hands and soles of his or her feet. The weakness spreads to the shoulders and the muscles of the face and the chest, making it hard to breathe.
- Your child will experience hot and cold flashes, including sweating and blushing.
- The pediatrician performs a series of tests including a spinal tap (a needle is inserted into your child’s spine to withdraw fluid for testing). There is a chance that your child will be hospitalized for several days and may need to be put on a breathing machine.
- After the child leaves the hospital, the muscle weakness and tingling or numbness problems continue and prevent your child from his/her normal activities for a few weeks to several months. The weakness and symptoms gradually get better over the next 12 months, but some weakness and symptoms may not completely go away.

Risk reduction for willingness-to-pay question: Chance of Guillain Barré Syndrome after vaccination from 1 in 1 million to No chance.

1. Descriptions were presented to respondents in the order seen in this appendix. [↑](#footnote-ref-2)
